# Biological relevance of Granzymes A and K during *E. coli* sepsis

**DOI:** 10.7150/thno.59418

**Published:** 2021-10-17

**Authors:** Iratxe Uranga-Murillo, Elena Tapia, Marcela Garzón-Tituaña, Ariel Ramirez-Labrada, Llipsy Santiago, Cecilia Pesini, Patricia Esteban, Francisco J Roig, Eva M Galvez, Phillip I Bird, Julián Pardo, Maykel Arias

**Affiliations:** 1 Fundación Instituto de Investigación Sanitaria Aragón (IIS Aragón), Biomedical Research Centre of Aragón (CIBA), 50009, Zaragoza, Spain.; 2 Animal Unit, University of Zaragoza, 50009, Zaragoza, Spain.; 3 Instituto de Carboquímica ICB-CSIC, 50018, Zaragoza, Spain.; 4 Department of Biochemistry and Molecular Biology, Biomedicine Discovery Institute, Monash University, 3800, Clayton VIC, Australia.; 5 Aragon I+D Foundation (ARAID), 50018, Zaragoza, Spain.; 6 Nanoscience Institute of Aragon (INA), University of Zaragoza, 50018, Zaragoza, Spain.; 7 Department of Microbiology, Preventive Medicine and Public Health, University of Zaragoza, 50009, Zaragoza, Spain.; 8 San Jorge University, 50830, Zaragoza, Spain.

**Keywords:** Granzyme K, Granzyme A, bacterial sepsis, inflammation

## Abstract

**Aims:** Recent *in vitro* findings suggest that the serine protease Granzyme K (GzmK) may act as a proinflammatory mediator. However, its role in sepsis is unknown. Here we aim to understand the role of GzmK in a mouse model of bacterial sepsis and compare it to the biological relevance of Granzyme A (GzmA).

**Methods:** Sepsis was induced in WT, GzmA^-/-^ and GzmK^-/-^ mice by an intraperitoneal injection of 2x10^8^ CFU from *E. coli*. Mouse survival was monitored during 5 days. Levels of IL-1α, IL-1β, TNFα and IL-6 in plasma were measured and bacterial load in blood, liver and spleen was analyzed. Finally, profile of cellular expression of GzmA and GzmK was analyzed by FACS.

**Results:** GzmA and GzmK are not involved in the control of bacterial infection. However, GzmA and GzmK deficient mice showed a lower sepsis score in comparison with WT mice, although only GzmA deficient mice exhibited increased survival. GzmA deficient mice also showed reduced expression of some proinflammatory cytokines like IL1-α, IL-β and IL-6. A similar result was found when extracellular GzmA was therapeutically inhibited in WT mice using serpinb6b, which improved survival and reduced IL-6 expression. Mechanistically, active extracellular GzmA induces the production of IL-6 in macrophages by a mechanism dependent on TLR4 and MyD88.

**Conclusions:** These results suggest that although both proteases contribute to the clinical signs of *E. coli*-induced sepsis, inhibition of GzmA is sufficient to reduce inflammation and improve survival irrespectively of the presence of other inflammatory granzymes, like GzmK.

## Introduction

Granzymes (Gzms) are a family of serine proteases classified according to their cleavage specificity. In humans, five different types of Gzms (A, B, H, K and M) have been described, while ten types have been described in mice (A, B, C, D, E, F, G, K, M and N) 1-3. It has been traditionally assumed that all Gzms act as cytotoxic proteases. However, recent evidence suggests that GzmB is the one with the greatest cytotoxic capacity, whereas the cytotoxicity of others such as GzmA and GzmK is in controversy 4. In addition, new evidences suggest that some Gzms like GzmA and GzmK may act as proinflammatory mediators 5.

GzmA and GzmK induce the generation of pro-inflammatory cytokines in different cell types. GzmA induces interleukin-1β, (IL-1β), tumour necrosis factor (TNFα) and interleukin-6 (IL-6) expression in human and mouse macrophages 6. Similarly, it has been demonstrated that human GzmK induces the expression of IL-6, IL-8 and the monocyte chemoattractant protein (MCP) in human lung fibroblasts and endothelial cells through a mechanism dependent on the activation of PAR-1 receptor 7, 8. Additionally, it has also been described that GzmK induce the generation of pro-inflammatory cytokines in mouse macrophages 9 and it potentiates LPS-induced cytokine responses in human monocytes 10.

Higher levels of GzmA and GzmK have been observed in serum from septic patients, suggesting that these proteases could play a role in the pathogenesis of sepsis 11, 12. The biological relevance of GzmA in sepsis has been demonstrated in different models of bacterial sepsis, including LPS-induced shock 6, 13, sepsis induced by the mouse bacterial pathogen *Brucella microti* 14, polymicrobial sepsis induced by caecal ligation and puncture 15 and pneumonia induced by *Streptococcus pneumoniae* 16. All these data suggest that GzmA plays an important role in bacterial sepsis. On the contrary, the role of GzmK in sepsis has not been studied yet *in vivo* due to the absence of mice deficient in this protease. Recently, Joeckel et al have generated GzmK deficient mice allowing the understanding of the biological relevance of GzmK 17. GzmK maps at the same chromosome locus as GzmA in both human and mice. Both, GzmA and GzmK, present similar cleavage specificity, preferentially cleaving substrates after basic residues like Arg and Lys. However, subtle differences have been recently found between the amino acid sequences close to the main cleavage site of GzmA and GzmK, which has been shown to influence the biological substrates cleaved by these proteases and likely their biological functions 18, 19.

Thus, it may be possible that GzmA and GzmK do not regulate the same biological functions *in vivo* and/or that they do not activate overlapping mechanisms. In order to understand the role of GzmK in the dysregulation of the inflammatory response in sepsis and the potential use of GzmA and/or GzmK as therapeutic targets in sepsis, it is critical to clarify the relative role of these Gzms in this pathology. Thus, the role of GzmK, in comparison with its closest homologue GzmA, in sepsis has been analysed using a mouse model of bacterial sepsis induced by the bacteria *E. coli,* one of the most common pathogens involved in human sepsis.

## Results

### Clinical score and survival are improved in the absence of GzmA

**Figure 1:**
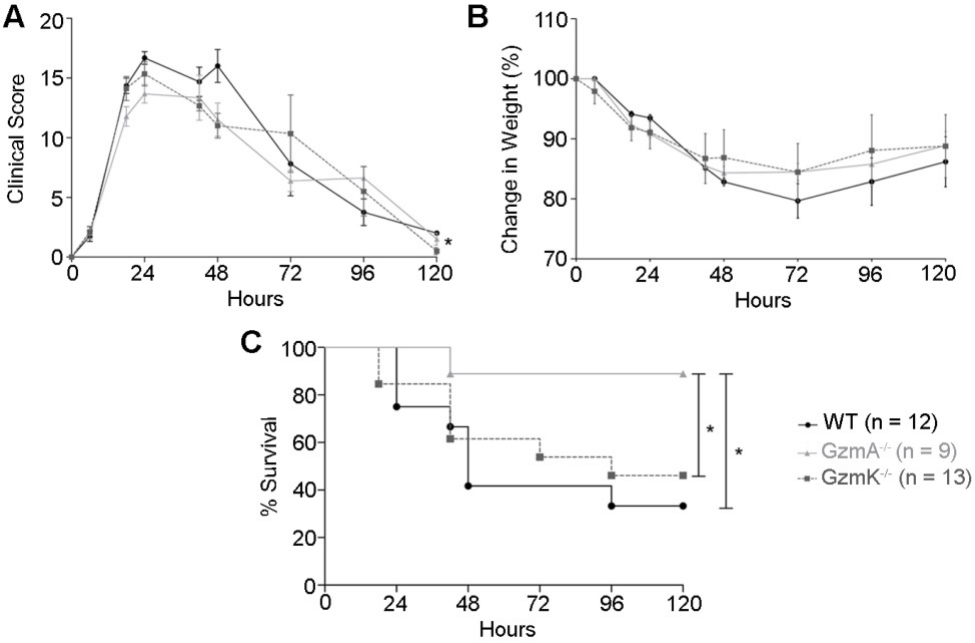
** Sepsis score, weight loss and survival during *E. coli*-induced sepsis.** WT, GzmA^-/-^ and GzmK^-/-^ mice were infected with 2 x 10^8^ CFU i.p. of *E*.* coli.* Animals were weighted daily; sepsis score was determined as described in materials and methods and survival was monitored for 5 days. **A.** Sepsis score. **B.** Changes in weight. Data are represented as mean ± SEM of sepsis score (A) or the mean ± SEM of the percent of weight loss (B). Statistical analyses were performed using a mixed linear regression as described in materials and methods. **C.** Percentage of survival. Statistical analysis was performed using logrank and Gehan-Wilcoxon test. In the figure the number of biological replicates from each group (n) of two independent experiments are indicated.

Sepsis was induced in WT, GzmA and GzmK deficient mice by i.p. administration of 2×10^8^ CF U/mL of an *E. coli* strain isolated from blood of mice suffering from CLP-induced sepsis. We employed this strategy in order to analyse sepsis induced by a mouse specific *E. coli* strain in order to avoid potential differences due to use non-species-specific strains. Sepsis score (Figure 1A), animal weight (Figure 1B), and survival (Figure 1C) were monitored for 5 days. All mice suffered a significant but similar loss of weight, which is a characteristic sign of sepsis (Figure 1B). In addition, mice showed others signs of sepsis such as piloerection, decreased movement, decreased respiratory rate, laboured breathing and a decreased response to auditory and tactile stimuli. These symptoms were quantified applying a murine sepsis score (MSS) as previously described 20. At 24 h of sepsis induction all the strains reached their highest sepsis score that was not significantly difference between WT and KO mouse strains. However, at 48 h of sepsis induction, GzmA^-/-^ and GzmK^-/-^ mice showed a slight but significant lower sepsis score compared with WT mice. A mixed linear regression analysis of this data showed significant differences between groups indicating that the absence of either GzmA or GzmK improved MSS (p = 0.035). In contrast to MSS, the covariance analysis showed that these differences were not due to a relation between MSS and weight (p = 0.366). The analysis of survival showed that despite the fact that GzmA^-/-^ and GzmK^-/-^ mice showed a similar loss of weigh and sepsis score, only GzmA deficient mice showed a significant increase in survival compared with WT mice. In contrast, GzmK deficient animals behaved as WT controls, beginning to die 48 h after sepsis induction and only 33% of WT and 46% of GzmK^-/-^ mice remained alive after 5 days. In contrast, 90% of GzmA^-/-^ animals survived to sepsis.

### *In vivo* bacterial replication is not affected by GzmA or GzmK deficiency

**Figure 2:**
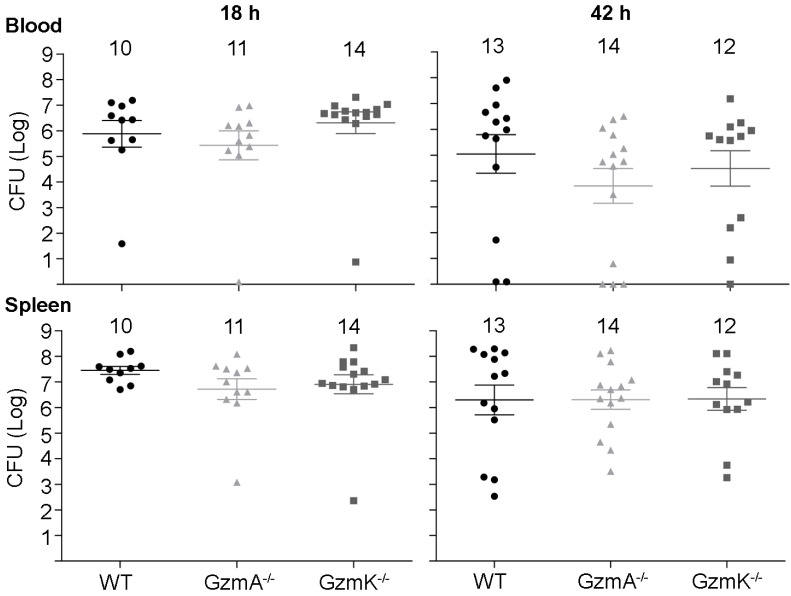
** Bacterial load in blood and spleen during *E. coli* sepsis.** WT, GzmA^-/-^ and GzmK^-/-^ mice were infected with 2 x 10^8^ CFU i.p. of *E*.* coli*. After 18 and 42 h of sepsis induction a group of animals was sacrificed and the bacterial load determined in blood and spleen. Data are represented as mean ± SEM CFU counts. Numbers at the top of each graph indicate the number of biological replicates from each group of two independent experiments. Statistical analysis was performed by one-way ANOVA test with Bonferroni's post-test.

Next, we analysed the role of GzmA and GzmK in the control of *E. coli* infection. As shown in Figure 2, WT, GzmA^-/-^ and GzmK^-/-^ mice showed a similar bacterial load in blood and spleen at both 18 and 42 h, time after which some animals had already begun to clear the infection. These results indicate that GzmA and GzmK are not involved in the control of *E. coli* infection and, thus, the increased survival of GzmA deficient mice is not due to differences in bacterial replication *in vivo*.

### Pro-inflammatory cytokines are reduced in GzmA deficient mice during *E. coli* sepsis

**Figure 3:**
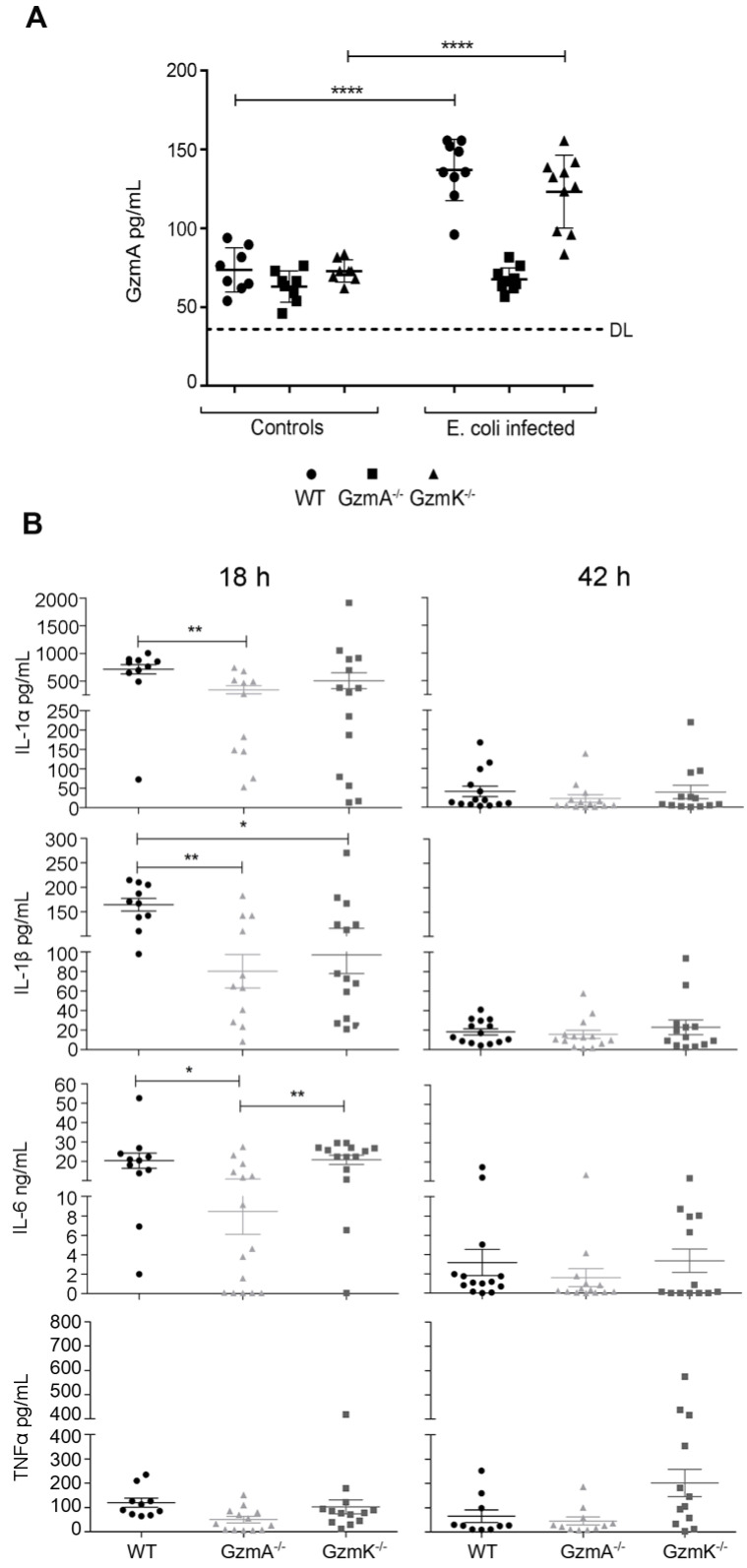
** Extracellular GzmA and proinflammatory cytokine levels in plasma during *E. coli* sepsis**. WT, GzmA^-/-^ and GzmK^-/-^ mice were infected with 2 x 10^8^ CFU i.p. of *E*.* coli*. After 18 and 42 h of sepsis induction a group of animals was sacrificed and the levels of GzmA (**A**), IL-1α, IL-1β, IL-6 and TNFα (**B**) in plasma were determined by ELISA. Data are represented as mean ± SEM of the values of each cytokine in 3 independent experiments. Statistical analysis was performed by one-way ANOVA test with Bonferroni's post-test *p < 0.05; **p < 0.01.

Once we had confirmed that the protection observed in GzmA^-/-^ mice was not related with the control of infection, we analyzed if extracellular GzmA was detected in serum from septic animals and if the absence of GzmA attenuated the inflammatory response in mice. As shown in Figure 3 at 18 h, extracellular GzmA in serum was significantly increased in WT and GzmK^-/-^ mice but, as expected, remained almost undetectable in GzmA deficient mice, which were included as controls to establish the cut-off of the ELISA test. GzmA deficient mice had lower levels of all tested cytokines in comparison with WT mice, although only IL-1α, IL-1β and IL-6 reached a statistically significant difference. Concerning GzmK deficient mice, they showed lower levels of IL-1β compared with WT mice. In addition, the level of inflammatory cytokines was also reduced in GzmA deficient mice compared with GzmK deficient, although only IL-6 was significantly different. After 42 h, most animals already presented very low level of cytokines in blood without differences between WT and mutant mice. We did not further analyse the level of cytokine expression since most mice died during the first 48 hours.

### GzmA and GzmK are mainly expressed in NK and NKT cells during sepsis

**Figure 4:**
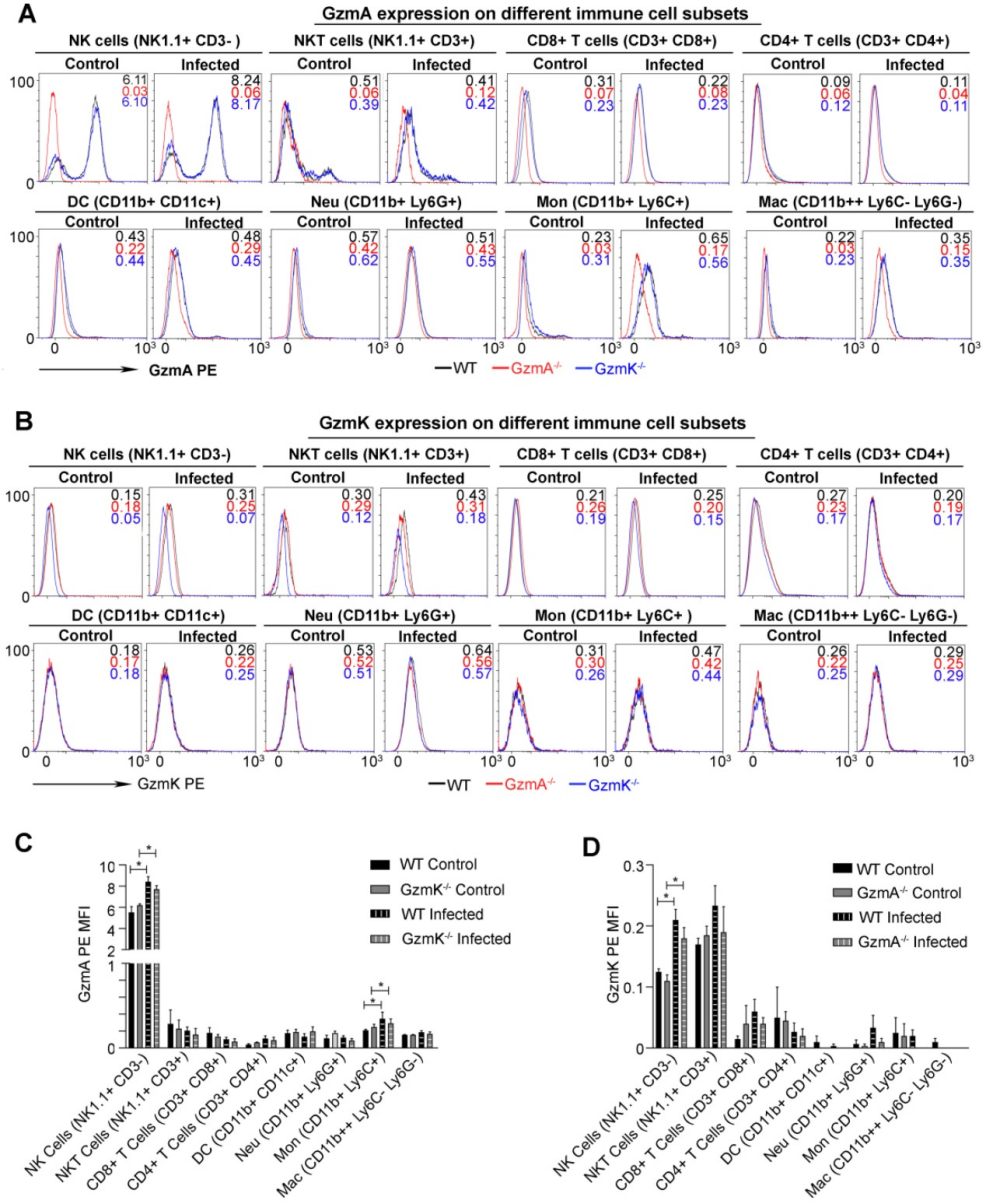
** Intracellular expression of GzmA and GzmK during *E. coli* sepsis.** WT, GzmA^-/-^ and GzmK^-/-^ mice were infected with 2 x 10^8^ CFU i.p. of *E. coli*. After 18 h of sepsis induction a group of animals were sacrificed and spleens were collected. The intracellular expression of GzmA and GzmK was analysed in splenocytes by flow cytometry as indicated in materials and methods. A representative histogram analysis is shown for GzmA (**A**) and GzmK (**B**). Numbers in the histograms show MFI (Mean Fluorescence Intensity) in each strain (WT in black, GzmA^-/-^ in red and GzmK^-/-^ in blue). Data in graphs represent the mean ± SEM of MFI for GzmA-PE (**C**) and GzmK-PE (**D**) minus the florescence of each granzyme deficient control mice in the immune cells subsets analyzed from three biological replicates. Statistical analysis was performed using one-way ANOVA test with Bonferroni's post-test. * p < 0.05.

Subsequently we analysed the cell source of GzmA and GzmK during sepsis induced by *E. coli*. We expected that the main cell source of GzmA in this model to be a component of the innate immune response since reduction of inflammation in GzmA^-/-^ and GzmK^-/-^ mice was observed within the first 24 h after infection. Intracellular expression of GzmA and GzmK was analysed in different immune spleen cell subsets 18 h after sepsis induction. As shown in Figure 4, NK and NKT cells increased the intracellular expression of GzmA and GzmK during *E. coli* sepsis in comparison with Gzm KO mice used as controls. In addition, we observed a slight increase in the expression of GzmA in monocytes (Ly6C+CD11b+Ly6G-CD11c-). As expected GzmA and GzmK followed a similar expression profile in GzmK and GzmA deficient mice, respectively, and they were not expressed in the corresponding deficient mice, confirming specific Gzm detection.

### Active GzmA induces the expression of IL-6 by a TLR4 and MyD88 dependent pathways

**Figure 5:**
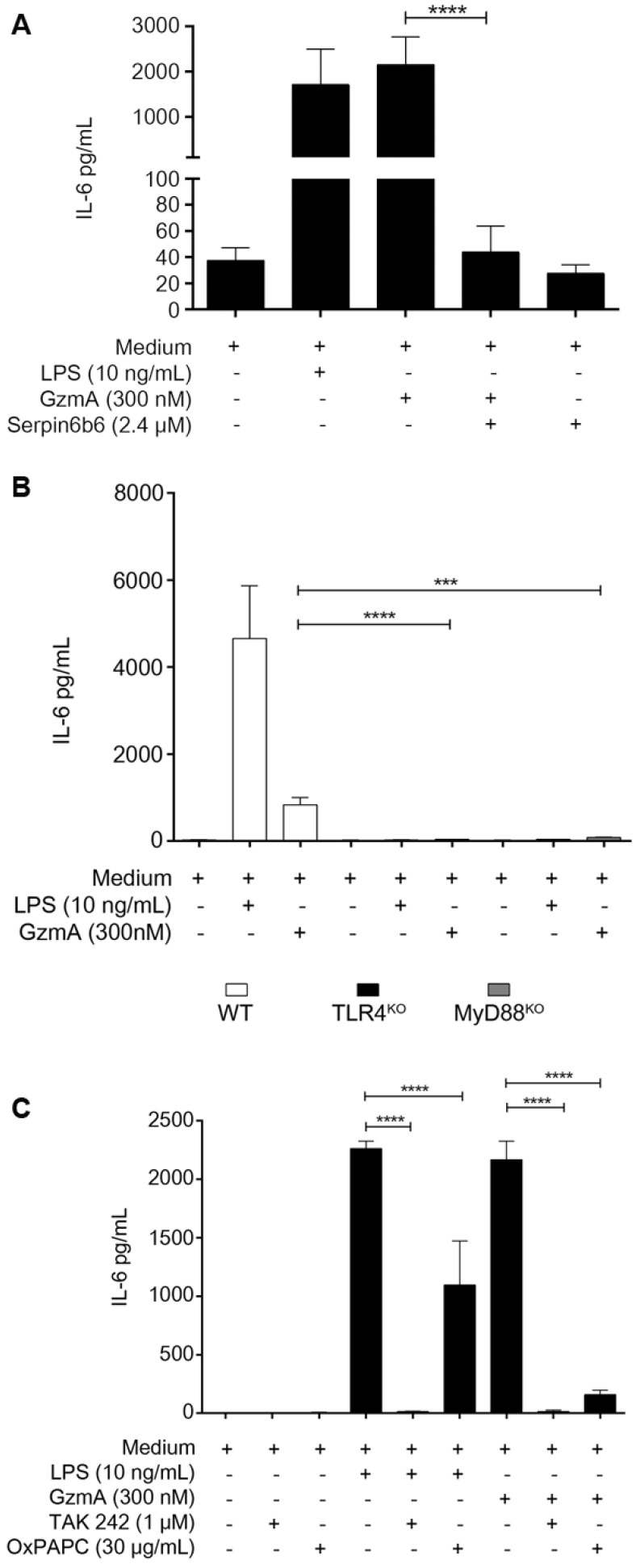
**Active granzyme A induces the expression of IL-6 by a TLR4 and MyD88 dependent pathway.** WT bone marrow differentiated macrophages were stimulated with LPS (10 ng/mL), active GzmA (300 nM) and GzmA inactivated with sepinb6b. After 24 h of incubation, supernatants were collected to determine the levels of IL-6 by ELISA (**A**). Macrophages differentiated from WT, TLR4^-/-^ and MyD88^-/-^ mouse bone marrow were stimulated with active GzmA (300 nM) and LPS (10 ng/mL). After 24 h of incubation, the supernatants were collected to determine the levels of IL-6 by ELISA (**B**). Data are represented as the mean ± SEM of two independent experiments performed by triplicate. Statistical analyses were performed by unpaired t test, ***p = 0.0001, ****p < 0.0001. WT bone marrow differentiated macrophages were first stimulated with TAK 242 (1 μM), OxPAPC (30 μg/mL) for 30 min at 37 °C and then stimulated with LPS 10 ng/mL and active GzmA (300 nM). After 24 h incubation, supernatants were collected to determine the levels of IL-6 by ELISA (**C**). Data are represented as the mean ± SEM of three independent experiments performed by triplicate. Statistical analyses were performed by unpaired t test ****p < 0.0001.

When added to M1 bone marrow-derived macrophages active GzmA was able to induce the expression of IL-6 which was inhibited by serpinb6b (Figure 5A). We have previously found that TLR4 is required for GzmA-induced inflammation in macrophages. In order to confirm this result and further get more insights into de potential mechanism activated by GzmA, the expression of IL-6 in GzmA-stimulated WT, TLR4^-/-^ and MyD88^-/-^ macrophages was analysed. As shown in figure 5B, IL-6 expression was significantly reduced in TLR4^-/-^ and MyD88^-/-^ macrophages compared to WT. Next, to delineate if GzmA activates specific TLR4 signalling pathways we used chemical inhibitors targeting different molecules involved in TLR4-mediated signalling. Specifically, we used TAK-242 and OxPAPC (Oxidized 1-palmitoyl-2-arachidonyl-sn- glycero-3-phosphorylcholine). TAK-242 is a small molecule inhibitor that blocks the intracellular TLR4 domain responsible of recruiting both TIRAP (MyD88 dependent signalling) and TRAM (MyD88 independent signalling) 21. OxPAPC is an oxidized phospholipid that inhibits LPS-mediated signalling by blocking its binding to LBP, CD14 and MD2, all of them adaptor molecules required for LPS to activate TLR4 signalling 22. As shown in Figure 5C, the expression of IL-6 induced by active GzmA was significantly reduced by both inhibitors. Together with the results using TLR4 and MyD88 deficient macrophages this result suggests that GzmA activates the conventional MyD88 and TIRAP dependent TLR4 signalling.

### Therapeutic inhibition of GzmA with serpinb6b improves survival and reduces inflammation in E. coli sepsis

**Figure 6:**
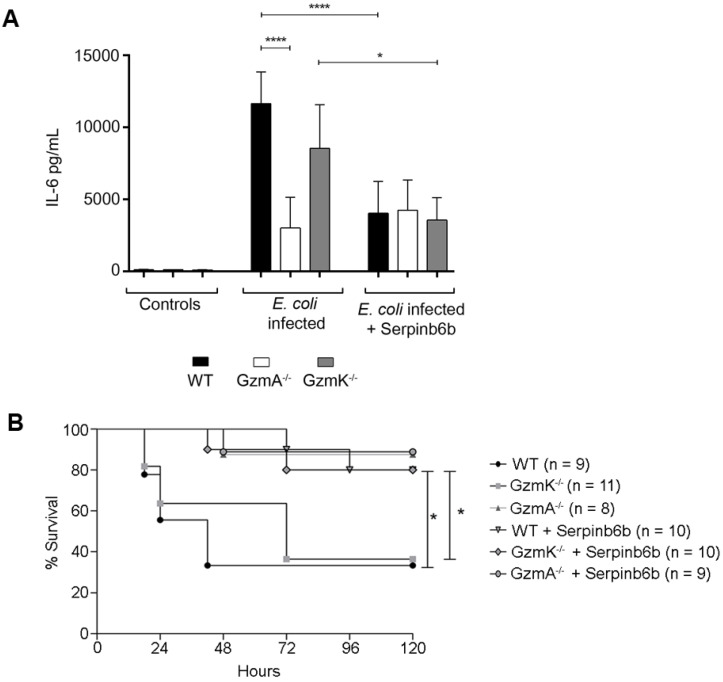
** Therapeutic inhibition of GzmA with serpinb6b during *E. coli* sepsis increase survival and reduces serum IL-6 in WT and GzmK deficient mice.** WT, GzmA^-/-^ and GzmK^-/-^ mice were infected with 2 x 10^8^ CFU i.p. of *E*.* coli.* After *E. coli* sepsis induction, a group of WT, GzmA and GzmK deficient mice were treated twice a day with 40 µg of serpinb6b in 100 µl of PBS i.p. for five days. As control a group of mice were treated with 100 µl of PBS i.p. twice a day. After 18 h a group of animals was sacrificed and IL-6 concentration in plasma was determined by ELISA (A). Data are represented as mean ± SEM of the values 2 independent experiments. Statistical analysis was performed by one-way ANOVA test with Bonferroni's post-test ****p < 0.0001. Mice were observed twice a day and survival was monitored for five days (B). Statistical analysis was performed using logrank and Gehan-Wilcoxon test. In the figure the number of biological replicates from each group (n) of two independent experiments are indicated. *p < 0.05; ***p < 0.001.

Finally, we analysed the effect of therapeutic inhibition of GzmA in *E. coli* sepsis. For that, after sepsis induction a group of WT, GzmA and GzmK deficient mice were treated with the specific GzmA inhibitor serpinb6b 23. As shown in Figure 6, only a 40% of WT and GzmK^-/-^ mice survived to *E. coli* sepsis. In contrast, when WT and GzmK^-/-^ were treated with serpinb6b, survival was significantly increased to 80%. In addition, similarly to the results obtained *in vitro* in GzmA-stimulated macrophages (Figure 5A), the levels of IL-6 in septic mice treated with serpinb6b were significantly reduced in WT and GzmK^-/-^ septic mice. While the levels of IL-6 in GzmA deficient mice treated with serpinb6b were not affected, confirming the specificity of this inhibitor. These results indicate that inhibition of GzmA is sufficient to improve sepsis outcome irrespectively of the presence of other inflammatory Gzms, like GzmK.

## Discussion

GzmA and GzmK have been shown to activate inflammatory responses in macrophages and other cell types 1 and increased levels of GzmA and GzmK have been detected in serum of septic patients suggesting that these proteases could have a role in bacterial sepsis 11, 12, 24. Here, using a mouse model of *E. coli* induced sepsis, the role of GzmK in an *in vivo* mouse model of bacterial sepsis has been analysed for the first time. In addition, the biological relevance of GzmA and GzmK in sepsis has been compared. Our results show that despite both proteases contribute to bacterial sepsis, including disease progression and generation of some inflammatory cytokines *in vivo*, only the absence of GzmA has a relevant impact in sepsis survival. In addition, it has been observed that neither GzmA nor GzmK are involved in the control of *E. coli* infection.

Both, GzmA and GzmK, were found to be mainly expressed in NK and NKT cells during *E. coli* sepsis. Both NK and NKT cells have been found to play important roles in bacterial sepsis 25-28 and together with T cells they have been shown to be the main cell sources of gzms *in vivo* 25-27. Except for monocytes that presented a slight increased expression of gzmA during sepsis, we did not find gzmA and/or gzmK in other cell types including T cells, macrophages, dendritic cells and neutrophils. It has been previously shown in other models that the presence of NK cells and GzmA is key for bacterial sepsis 14 and endotoxicosis 13. Although we have not formally proven the role of NK and NKT cells in *E. coli* sepsis, our results together with previous data strongly suggest that this cell type is an important source of Gzms during *E. coli* sepsis.

During sepsis induced by a commensal strain of *E. coli* isolated from blood of a mice suffering from sepsis induced by CLP, a lower sign of sepsis was observed in GzmA or GzmK deficient mice, when a murine sepsis score was applied, compared to WT mice. This murine sepsis score has been validated by Shrum et al and analyse several parameters such as respiratory function, animal behaviour and response to stimuli, allowing the evaluation of sepsis progression in mice 20. However, only GzmA deficient mice showed a significant increase in survival compared with WT mice. In addition, GzmA deficient mice survived significantly more than GzmK deficient mice, suggesting that GzmA has a higher biological relevance than GzmK during *E. coli* sepsis. It is worth to mention again that we have employed an *E. coli* strain isolated from mice undergoing polymicrobial peritoneal sepsis (CLP) and, thus, our results are biologically relevant since a species-specific bacterial pathogen is employed.

These results employing a mouse specific *E. coli*, one of the most common pathogens causing sepsis in human, confirm previous studies using other models on the inflammatory function of GzmA *in vitro* and *in vivo* during host-pathogen interaction. It has been previously found that GzmA deficient mice are resistant to endotoxemia induced by LPS 6, 13. In addition, GzmA deficient mice showed an increase in survival compared with WT mice during sepsis induced by the mouse specific pathogen *B. microti* 14*.* The increase in survival was correlated with lower levels of proinflammatory cytokines IL-1α, IL-1β, TNFα and IL-6. GzmA was also found to be not involved in bacterial control. It has been recently shown that GzmA plays an important role in the damage associated with *S. pneumoniae* infection, the most common causative agent in community-acquired pneumonia 29. In addition, GzmA deficient mice also showed higher survival in a model of pneumonia due to *S. pneumoniae* 16. In our study, GzmA deficient mice, in addition to a higher survival, express lower levels of IL-1α, IL-β and IL-6 in serum compared with WT mice. These cytokines have been involved in the physiopathology of bacterial sepsis 30-32 and the lower levels of these cytokines in GzmA deficient mice could explain their higher survival during *E. coli* sepsis. However, it has also been observed that in murine models of *Klebsiella pneumoniae* pneumonia, a common pathogen in intrahospital pneumonias, GzmA does not play an important role during sepsis associated with this pathogen 33. A recent study has not been able to confirm a clear role for GzmA or GzmB during *E. coli* sepsis 34. However, the results obtained were not consistent and a clear conclusion on the role of GzmA and/or GzmB in bacterial control and sepsis could not be reached. Notably, a main difference among both studies is the use of different *E. coli* strains. Meanwhile an *E. coli* strain isolated from humans was used by Garcia-Laorden et al, we have employed a bacterial strain isolated from the blood of septic mice. Therefore, the biological relevance of this protease in sepsis seems to be dependent on the causal agent of the infection.

Although the impact of GzmK absence in *E. coli* sepsis seems to be less pronounced than that of GzmA absence, GzmK deficient mice have shown significantly lower levels of IL-1β in serum compared with WT mice. This result could explain the lower sepsis score observed in GzmK deficient mice during *E. coli* induced sepsis. This is the first time that the role of GzmK in bacterial sepsis is analysed *in vivo*. These findings are supported by previous *in vitro* findings indicating that GzmK may have a prominent role in regulating IL-1β function. Using recombinant GzmK, it has been demonstrated that GzmK induces the expression of IL-1β in peritoneal macrophages pre-stimulated with LPS 9. Recently, it has also been observed that human GzmK is able to potentiate LPS-induced release of pro-inflammatory cytokines in human monocytes 10. In a recent work, Wensink et al, reported that similarly to GzmK, human GzmA also potentiates cytokine responses in human monocytes pre-stimulated with LPS or Gram-negative bacteria. They found that contrary to GzmK, GzmA does not bind to LPS or increase the LPS-CD14 complex formation and has a little effect on LPS micelle disaggregation, concluding that GzmA and GzmK differentially modulate LPS-Toll-like receptor signalling in monocytes 35. This result suggests that GzmA and GzmK have not a redundant function in antibacterial immune response and help to explain the differences observed in our study. Indeed, in contrast to GzmK, recent evidences indicate that GzmA induces IL-6 production in mouse macrophages independently of LPS 15. In line with these previous results, we show here that active GzmA induces the expression of IL-6 by a mechanism dependent of TLR4. In this study we have further analysed the role of TLR4 signalling during gzmA-induced IL-6 production in macrophages by using cells derived from MyD88 deficient mice and inhibitors targeting different molecules involved in TLR4 signalling, finding out that IL-6 production induced by gzmA in mouse macrophages mainly depends on the activation of TLR4 by the canonical MyD88 pathway.

We have found that similarly to GzmA, GzmK did not play an important role in the control of *E. coli* infection. This is the first time that the role of GzmK in the control of bacterial infection is analysed. Our finding is in line with those of Joeckel et al., showing that GzmK deficient mice controlled viral infections as efficiently as WT mice 17. However, further research is needed to confirm the role of inflammation induced by GzmK in the control of other bacterial infections, which is not the main aim of this work. Our results have confirmed that in contrast to GzmA, GzmK has a minor role during *E. coli* sepsis. Apparently, this result might be unexpected since both GzmA and GzmK are proteases with tryptase activity and share preferences to cleave substrates after basic residues (Lys or Arg). However, it has been shown that despite the similar protease activity, the substrate specificity of GzmK differs from GzmA 18, 19 , which would explain the different contribution of these proteases to sepsis.

Despite we have not found a major role of GzmK in sepsis, our findings are important since they suggest that the detrimental role of GzmA during *E. coli* sepsis cannot be significantly compensated by its closest homologue GzmK and, thus, inhibition of GzmA might be sufficient to reduce damage and increase survival during sepsis. Indeed, our results show that therapeutical inhibition of active GzmA with serpinb6b similarly increased survival in WT and GzmK ^-/-^ mice and reduced IL-6 serum levels, confirming the potential of GzmA as a therapeutic target for sepsis treatment 15.

In conclusion, the role of GzmK in an *in vivo* mouse model of bacterial sepsis has been analysed for the first time and the biological relevance of GzmK and GzmA has been compared in bacterial sepsis. Both proteases have been found to be involved in physiopathology of bacterial sepsis. However, GzmA seems to play a more relevant role in this pathology and targeting GzmA seems to be sufficient to reduce inflammation and increase survival in sepsis. It will be required to analyse other models of sepsis *in vivo* including single and polymicrobial to confirm if GzmK is a minor regulator during bacterial sepsis, and, thus, design proper protocols to use the Gzm family as new therapeutic targets in sepsis.

## Materials and methods

### Mouse Strains

Inbred C57BL/6 (WT), Granzyme A deficient (GzmA^-/-^)^36^ and Granzyme K deficient mouse strains (GzmK^-/-^) 17, 37 were maintained at the Biomedical Research Centre of Aragon (CIBA). Both, GzmA and GzmK deficient mice are on the C57BL/6 background and their genotypes were periodically analysed as described 17, 37. Mice of 8-12 weeks of age were used in all the experiments. Animal experimentation protocols were approved by the CIBA Animal Experimentation Ethics Committee (number: PI63/17).

### *E. coli* sepsis induction

An *E. coli* strain was isolated from blood of wild type C57Bl76 mice after 24 h of sepsis induced by cecal ligation puncture (CLP) procedure. This strain was the most frequent Gram-negative bacteria isolated from blood of this septic mouse. The *E. coli* strain was stored at -80 °C in Luria-Bertani medium (LB; Sigma) containing 10% glycerol. To prepare the inoculum for sepsis induction, 10 µl of *E. coli* stock was cultured in LB medium at 37 °C to exponential growth phase and washed twice with cold phosphate-buffered saline (PBS). The absorbance at 600 nm was measured in a spectrophotometer to estimate the number of bacteria in the culture. The bacterial density was adjusted to 1x10^9^ bacteria/mL. Sepsis was induced in WT, GzmA^-/-^ and GzmK^-/-^ mice by intraperitoneal injection of 2×10^8^ bacteria in 200 µl of PBS.

### Survival experiment and Sepsis Score

Mice were weighed daily and observed twice a day determining a murine sepsis score (MSS) as described by Shrum et al. 20. Briefly, seven variables were analysed (Appearance, Level of consciousness, Activity, Response to stimulus, Eyes, Respiration rate and Respiration quality), each one received a score between 0 and 4. Mice were euthanized when MSS was superior to 21, or when either the score of Respiration rate or Respiration quality increased more than 3. Survival was monitored for 5 days.

### Determination of Bacterial load in blood, liver and spleen

A group of mice were sacrificed 24 and 48 hours after sepsis induction, and blood, liver and spleen were collected aseptically. Both organs were homogenised, and serial dilutions were carried out in PBS, including the blood, plated onto LB agar and incubated for 24 h at 37 °C to determine the number of viable *E. coli* organisms.

### Determination of cytokine and GzmA A levels in plasma

24 and 48 hours after sepsis induction, a group of mice was sacrificed and blood samples were collected by cardiac puncture using anticoagulant. Blood samples were centrifuged at 3700 xg to obtain plasma. Levels of IL-1α, IL-1β, TNFα and IL-6 in plasma were measured using commercial ELISA kits (Invitrogen) and GzmA levels were measured using Mouse Granzyme A ELISA kit (abcam), following the manufacturer recommendations.

### Inflammation induced by GzmA in macrophages from WT, TLR4^-/-^ and MyD88^-/-^ mice

M1 macrophages were differentiated from mouse bone marrow (BM) of WT, TLR4^-/-^ and MyD88^-/-^ mice. Cells were aseptically collected from femurs and tibias and resuspended in RPMI medium supplemented with 10% of FCS serum, 100 U/mL of penicillin/streptomycin, 50 mM of 2-ME, and 10% supernatant of X-63 cell culture as source of GM-CSF. Cells were seeded at a density of 1 × 10^6^ cells/mL and allowed to differentiate for 6 days at 37 °C and 5% CO2 atmosphere. For *in vitro* experiments, 5 x 10^5^ macrophages were seeded in 96 well plates and incubated with 300 nM of active GzmA overnight at 37 °C and 5% CO2. After incubation, supernatants were collected and IL-6 levels were measured using a commercial ELISA kit. In some cases, the TLR4 pathway inhibitors TAK242 (Merck) (1 μM) and OxPAPC (InvivoGen) (30 μg/mL) were used in WT macrophages. Cells were preincubated with the inhibitors for 30 min at 37 °C/5%CO2 and, subsequently, treated with LPS (10 ng/mL) or active GzmA (300 nM) overnight at 37 °C/5%CO2. After incubation, supernatants were collected and IL-6 was measured using a commercial ELISA kit (Invitrogen).

### Intracellular expression of GzmA and GzmK

After 18 h of sepsis induction, WT, GzmA^-/-^ and GzmK^-/-^ mice were sacrificed. Spleens were collected aseptically, homogenized in 5 mL of RPMI medium and then erythrocytes were lysed. For analysis of GzmA and GzmK expression, 1×10^6^ splenocytes were stained with extracellular fluorescent labelled antibodies anti CD45-BV421, anti CD3-FITC, anti CD8-APC, and anti NK1.1-APC-Vio770 for NK cells, NKT cells and CD8 lymphocytes characterization. Anti CD3-FITC and anti CD4-VioGreen for CD4 lymphocytes. And Ly6G-FITC, Ly6C-VioGreen, CD11b-APC-Vio770, CD11c-APC for macrophages, monocytes, neutrophils and dendritic cells. All antibodies used were from Miltenyi Biotec. Subsequently, cells were fixed with paraformaldehyde (PFA) 1%, then permeabilized with saponin 1% in PBS 5% FBS and incubated with intracellular PE conjugated anti GzmA (eBioscience) and PE conjugated anti GzmK (MyBioSource). Finally, expression of GzmA was analyzed by FACS. The gating strategy was the following: First, doublets (FSC-H vs FSC-A) and debris (SSC-H vs. SSC-A) were excluded. Next, SSC-A vs. CD45 gating was done to identify CD45+ cells population. From the CD45+ population, NK cell, NKT cell, CD8 and CD4 T lymphocyte, macrophage, monocyte, neutrophil and dendritic cell populations were identified. Finally, GzmA and GzmK expression was analyzed in each cell population.

### Therapeutic Inhibition of GzmA with serpinb6b during *E. coli* sepsis

After *E. coli* sepsis induction, a group of WT, GzmA and GzmK deficient mice were treated twice a day with 40 µg of serpinb6b, a specific mouse GzmA inhibitor, in 100 µl of PBS i.p. (intraperitoneal) for five days. As control a group of these mice were treated with 100 µl of PBS i.p. twice a day. Mice were observed twice a day and survival was monitored for five days.

### Recombinant mouse serpinb6b expression from Pichia pastoris

Pichia pastoris expressing serpinb6b was kindly provided by Phil Bird from Monash University, Australia. P. pastoris expressing serpinb6b was grown at 30 °C for 48 h; growth medium was replaced with induction medium containing 3% methanol and 0.5 M Arginine. Induction was performed at 30 °C for 72 h. Culture was centrifuged and a chemical and physical lysis was performed. After supernatant clarification, recombinant serpinb6b was purified by immobilized metal (Nickel) affinity chromatography.

### Statistical Analysis

To analyse the differences in MSS and weight between mouse groups we performed a Mixed Linear Regression with the IBM SPSS Statistics 25 software. MSS was chosen as the dependent variable, mouse group (WT, GzmA^-/-^ or GzmK^-/-^) as subject (grouping element), time as a factor and weight as a covariable. Differences between single groups were performed using ANOVA and Bonferroni´s post-test for the variable that showed statistical significances in Mixed Linear Regression. Logrank and Gehan-Wilcoxon test for survival and GzmA therapeutic inhibition analyses, one-way ANOVA test with Bonferroni's post-test for bacterial load in blood and spleen analysis and extracellular expression of GzmA and unpaired t test for inflammation induced by GzmA and TLR4 pathway inhibition experiments were performed with the GraphPad Prism software.

## Supplementary Material

Supplementary figure.

## References

[1] Arias M, Martinez-Lostao L, Santiago L, Ferrandez A, Granville DJ, Pardo J (2017). The Untold Story of Granzymes in Oncoimmunology: Novel Opportunities with Old Acquaintances. Trends cancer.

[2] Pardo J, Aguilo JI, Anel A, Martin P, Joeckel L, Borner C (2009). The biology of cytotoxic cell granule exocytosis pathway: granzymes have evolved to induce cell death and inflammation. Microb Infect / Institut Pasteur.

[3] Voskoboinik I, Whisstock JC, Trapani JA (2015). Perforin and granzymes: function, dysfunction and human pathology. Nat Rev Immunol.

[4] Joeckel LT, Bird PI (2014). Are all granzymes cytotoxic *in vivo*?. Biol Chem.

[5] Martinez-Lostao L, Anel A, Pardo J (2015). How Do Cytotoxic Lymphocytes Kill Cancer Cells?. Clin Cancer Res: an official journal of the American Association for Cancer Research.

[6] Metkar SS, Menaa C, Pardo J, Wang B, Wallich R, Freudenberg M (2008). Human and mouse granzyme A induce a proinflammatory cytokine response. Immunity.

[7] Sharma M, Merkulova Y, Raithatha S, Parkinson LG, Shen Y, Cooper D (2016). Extracellular granzyme K mediates endothelial activation through the cleavage of protease-activated receptor-1. FEBS J.

[8] Cooper DM, Pechkovsky DV, Hackett TL, Knight DA, Granville DJ (2011). Granzyme K activates protease-activated receptor-1. PloS one.

[9] Joeckel LT, Wallich R, Martin P, Sanchez-Martinez D, Weber FC, Martin SF (2011). Mouse granzyme K has pro-inflammatory potential. Cell Death Differ.

[10] Wensink AC, Kemp V, Fermie J, Garcia Laorden MI, van der Poll T, Hack CE (2014). Granzyme K synergistically potentiates LPS-induced cytokine responses in human monocytes. PNAS USA.

[11] Lauw FN, Simpson AJ, Hack CE, Prins JM, Wolbink AM, van Deventer SJ (2000). Soluble granzymes are released during human endotoxemia and in patients with severe infection due to gram-negative bacteria. J Infect Dis.

[12] Rucevic M, Fast LD, Jay GD, Trespalcios FM, Sucov A, Siryaporn E (2007). Altered levels and molecular forms of granzyme k in plasma from septic patients. Shock.

[13] Anthony DA, Andrews DM, Chow M, Watt SV, House C, Akira S (2010). A role for granzyme M in TLR4-driven inflammation and endotoxicosis. J Immunol.

[14] Arias MA, Jimenez de Bagues MP, Aguilo N, Menao S, Hervas-Stubbs S, de Martino A (2014). Elucidating sources and roles of granzymes A and B during bacterial infection and sepsis. Cell rep.

[15] Garzón-Tituaña M, Sierra-Monzón JL, Comas L, Santiago L, Paño-Pardo JR, Galvez EM (2020). Granzyme A inhibition reduces inflammation and increases survival during abdominal sepsis. Theranostics Accepted.

[16] van den Boogaard FE, van Gisbergen KP, Vernooy JH, Medema JP, Roelofs JJ, van Zoelen MA (2016). Granzyme A impairs host defense during Streptococcus pneumoniae pneumonia. Am J Physiol Lung Cell Mol Physiol.

[17] Joeckel LT, Allison CC, Pellegrini M, Bird CH, Bird PI (2017). Granzyme K-deficient mice show no evidence of impaired antiviral immunity. Immunol Cell Biol.

[18] Bovenschen N, Quadir R, van den Berg AL, Brenkman AB, Vandenberghe I, Devreese B (2009). Granzyme K displays highly restricted substrate specificity that only partially overlaps with granzyme A. J Biol Chem.

[19] Plasman K, Demol H, Bird PI, Gevaert K, Van Damme P (2014). Substrate specificities of the granzyme tryptases A and K. J Proteome Res.

[20] Shrum B, Anantha RV, Xu SX, Donnelly M, Haeryfar SM, McCormick JK (2014). A robust scoring system to evaluate sepsis severity in an animal model. BMC Res Notes.

[21] Matsunaga N, Tsuchimori N, Matsumoto T, Ii M (2011). TAK-242 (resatorvid), a small-molecule inhibitor of Toll-like receptor (TLR) 4 signaling, binds selectively to TLR4 and interferes with interactions between TLR4 and its adaptor molecules. Mol Pharmacol.

[22] Mackman N (2003). How do oxidized phospholipids inhibit LPS signaling?. Arterioscler Thromb Vasc Biol.

[23] Kaiserman D, Stewart SE, Plasman K, Gevaert K, Van Damme P, Bird PI (2014). Identification of Serpinb6b as a species-specific mouse granzyme A inhibitor suggests functional divergence between human and mouse granzyme A. J Biol Chem.

[24] Zeerleder S, Voves C, Wuillemin WA (2005). Comment on hemostatic markers and the sepsis-related organ failure assessment score in patients with disseminated intravascular coagulation in an intensive care unit by Okabayashi et al. Am J Hematol.

[25] Etogo AO, Nunez J, Lin CY, Toliver-Kinsky TE, Sherwood ER (2008). NK but not CD1-restricted NKT cells facilitate systemic inflammation during polymicrobial intra-abdominal sepsis. J Immunol Res.

[26] de Pablo R, Monserrat J, Torrijos C, Martin M, Prieto A, Alvarez-Mon M (2012). The predictive role of early activation of natural killer cells in septic shock. Crit Care.

[27] Kerr AR, Kirkham LA, Kadioglu A, Andrew PW, Garside P, Thompson H (2005). Identification of a detrimental role for NK cells in pneumococcal pneumonia and sepsis in immunocompromised hosts. Microb Infect/Institut Pasteur.

[28] Szabo PA, Anantha RV, Shaler CR, McCormick JK, Haeryfar SM (2015). CD1d- and MR1-Restricted T Cells in Sepsis. Front Immunol.

[29] van der Poll T, Opal SM (2009). Pathogenesis, treatment, and prevention of pneumococcal pneumonia. Lancet.

[30] Vanden Berghe T, Demon D, Bogaert P, Vandendriessche B, Goethals A, Depuydt B (2014). Simultaneous targeting of IL-1 and IL-18 is required for protection against inflammatory and septic shock. Am J Respir Crit Care Med.

[31] Chong DL, Sriskandan S (2011). Pro-inflammatory mechanisms in sepsis. Contrib Microbiol.

[32] Mirzarahimi M, Barak M, Eslami A, Enteshari-Moghaddam A (2017). The role of interleukin-6 in the early diagnosis of sepsis in premature infants. Pediatr Rep.

[33] Garcia-Laorden MI, Stroo I, Blok DC, Florquin S, Medema JP, de Vos AF (2016). Granzymes A and B Regulate the Local Inflammatory Response during Klebsiella pneumoniae Pneumonia. J Innate Immun.

[34] Garcia-Laorden MI, Stroo I, Terpstra S, Florquin S, Medema JP, van TVC (2017). Expression and Function of Granzymes A and B in Escherichia coli Peritonitis and Sepsis. Mediators Inflamm.

[35] Wensink AC, Kok HM, Meeldijk J, Fermie J, Froelich CJ, Hack CE (2016). Granzymes A and K differentially potentiate LPS-induced cytokine response. Cell death Discov.

[36] Ebnet K, Hausmann M, Lehmann-Grube F, Mullbacher A, Kopf M, Lamers M (1995). Granzyme A-deficient mice retain potent cell-mediated cytotoxicity. EMBO J.

[37] Pardo J, Wallich R, Martin P, Urban C, Rongvaux A, Flavell RA (2008). Granzyme B-induced cell death exerted by *ex vivo* CTL: discriminating requirements for cell death and some of its signs. Cell Death Differ.

