# Supplementary material for: Biological relevance of Granzymes A and K during E. coli sepsis
Source: Theranostics. 2021 Oct 17;11(20):9873–83. doi: 10.7150/thno.59418 (PMC8581435; doi:10.7150/thno.59418)
Supplement: Supplementary file 1 — Supplementary figure. [file thnov11p9873s1.pdf]

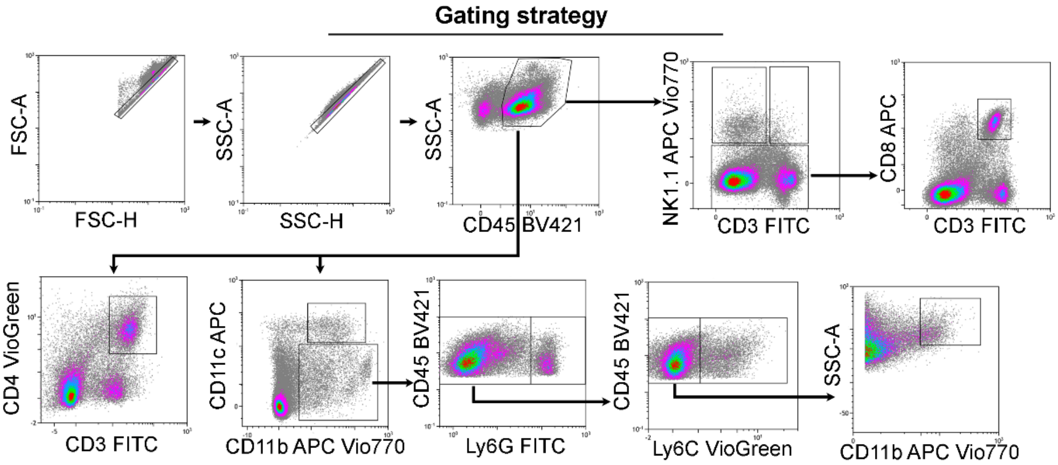

Supplementary figure 1. Gating strategy for GzmA and GzmK expression analysis. After 18 h of sepsis induction, WT, $GzmA^{-/-}$ and $GzmK^{-/-}$ mice were sacrificed. Spleens were collected aseptically, homogenized in 5 mL of RPMI medium and then erythrocytes were lysed. For analysis of GzmA and GzmK expression, $1x10^6$ splenocytes were stained with extracellular fluorescent labelled antibodies. Doublets (FSC-H vs FSC-A and SSC-H vs. SSC-A) were excluded. SSC-A vs. $CD45^+$ gating was done to identify $CD45^+$ cells population. For the first cocktail 3 subpopulations were identified: NK cell (NK1.1+CD3-), NKT cell (NK1.1+CD3+) and CD8 lymphocyte (CD8+CD3+) which were gated from NK1.1 negative cells. For the second cocktail CD4 T lymphocyte (CD4+CD3+) subpopulation was identified. Finally, for the third cocktail 4 subpopulations were identified: dendritic cell (CD11b+CD11c+), from CD11b+CD11c- cells a Ly6G+ population was identified as neutrophils (CD11b+Ly6G+). Next, from Ly6G- population a Ly6C+ population was identified as monocytes (CD11b+Ly6C+Ly6G-). Finally, from Ly6C- population a CD11b++ population was identified as macrophages (CD11b++Ly6C-Ly6G-).
